# The development of metastatic meningioma in a canine patient post-immunotherapy case report

**DOI:** 10.3389/fvets.2025.1646793

**Published:** 2026-01-05

**Authors:** Mercy Paine, Tamara Chamberlin, Jennifer Buczek, Jay McDonnell, Mary White

**Affiliations:** 1 Department of Pathology and Population Medicine, College of Veterinary Medicine, Midwestern University, Glendale, AZ, United States; 2 Veterinary Radiation Oncology of the Chesapeake, Annapolis, MD, United States

**Keywords:** meningioma, immunotherapy, metastasis, seizure, neoplasm, canine, case report

## Abstract

Metastatic meningioma is a rare occurrence in canine patients, with only four previous cases reported. This case report examines a recurrent meningioma with pulmonary metastasis in a male Labradoodle that survived 28 months post clinical presentation. The meningioma recurred following surgery and treatment with an autologous tumor cell lysate vaccine. This case explores the potential role of immunotherapy, by extending survival time, creating conditions that allow for the rare development of metastatic disease.

## Introduction

Meningiomas are among the most frequently diagnosed intracranial tumors in both human and veterinary medicine, particularly in dogs (1). Despite their prevalence, metastasis of these tumors remains rare—reported in less than 1% of human cases (2) and in only a handful of canine cases (3, 4). While meningiomas are typically considered benign, recurrence following resection—especially in higher-grade tumors—is a significant risk factor for metastasis in human patients (2). In veterinary medicine, treatment often centers on surgical resection, with or without adjunctive radiation therapy, which has been shown to significantly improve survival times (5–8). However, the prognosis for canine patients still lags behind that of their human counterparts.

This case report describes an unusual presentation of a canine meningioma with pulmonary metastasis, managed through a combination of surgical resection and experimental autologous immunotherapy. The patient experienced a survival time of 28 months—comparable and sometimes exceeding average outcomes reported in most literature with conventional treatment (5–7) —highlighting the potential of immunotherapeutic approaches to extend both quality and duration of life. Additionally, the concurrent development of a pancreatic adenocarcinoma in this geriatric patient raises questions regarding tumor biology, treatment side effects, and the role of age-related risk factors.

### Case description

An 11-year-old male Labradoodle presented on November 24, 2018, to his primary veterinarian for abnormal behavior over the preceding 24 h and suspected seizure activity. While at the clinic, the patient suffered a generalized tonic clonic seizure that was minimally responsive to diazepam and required administration of propofol. He was initially transferred to an emergency critical care facility and subsequently referred to Veterinary Neurology & Imaging of the Chesapeake for specialized care.

**Figure 1:**
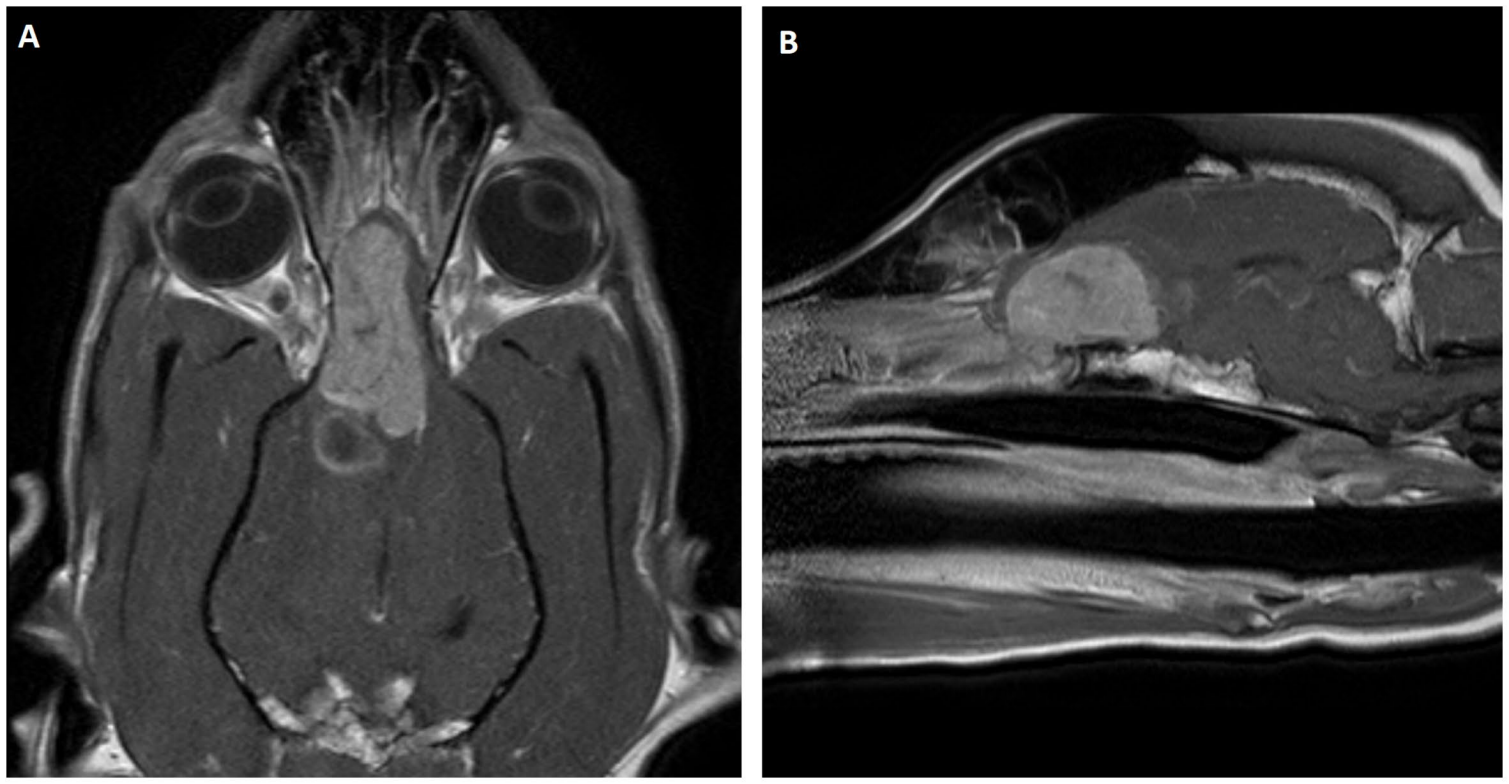
Magnetic Resonance Images (MRI) of the meningioma within the right falx cerebri compressing the right frontal olfactory bulb. T1 weighted images with contrast **(A)** is coronal and **(B)** is sagittal. A large contrast enhancing mass within the right olfactory and frontal lobes extending caudally to the level of the lateral ventricles. The mass is heterogeneously contrast enhancing. It measures 4.6 × 2.5 × 2.0 cm (rostrocaudally x dorsoventrally x transversely) there is mass effect with accompanying edema and central hemorrhage.

At the referral facility, neurological examination revealed cranial nerve deficits, left-sided vision loss, reduced and delayed postural reactions, left-sided ataxia, and dull mentation. An MRI performed on November 26, 2018, revealed a 4.6 × 2.5 × 2.0 cm mass in the right falx cerebri, compressing the right frontal olfactory bulb (Figure 1). The mass was initially suspected to be intra-axial. The patient was managed with prednisone and phenobarbital between diagnosis and surgery.

A transfrontal craniotomy was performed on December 18, 2018, along the right side of the frontal/olfactory bulb. Ultrasound was used to localize the abnormal tissue and associated cyst. An incision was made in the dura, and the mass along with the affected brain tissue was resected. The incision was closed with Gelfoam, and a fat graft was placed over the craniotomy site in the frontal sinus. The outer frontal bone was closed using polypropylene mesh.

The patient appeared to be recovering postoperatively, but began to decline 24 h later. A CT scan on December 20, 2018, revealed pneumocephalus, prompting a revision surgery to remove the air and reseal the frontal sinus and olfactory bulb. The patient then remained hospitalized for five more days before discharge.

At discharge, the patient’s neurological status was quiet but responsive to stimuli. He exhibited mild tetraparesis, right-sided pacing and circling, mild proprioceptive deficits in the left limbs, and an abnormal menace response in the left eye. He was discharged on prednisone, gabapentin, and phenobarbital as maintenance therapy.

Histopathological evaluation of the resected tissue showed polygonal neoplastic cells arranged predominantly in islands and whorls, with some sheeting. There was moderate mitotic activity, anisocytosis, and anisokaryosis, with large areas of necrosis present. No true invasion of neuroparenchyma was observed. The tumor was diagnosed as a meningothelial meningioma. However, the presence of necrosis, a sheet-like pattern, and moderate mitotic index warranted classification as a Grade II atypical meningioma (9).

Following surgery, the patient received immunotherapy with an autologous tumor cell lysate vaccine combined with toll-like receptor ligands. The vaccine was prepared by the University of Minnesota (10) using tumor tissue collected during surgery. The vaccine protocol, including the use of imiquimod, is described in Anderson et al. (10).

At the postoperative exam on December 31, 2018, the patient’s vision was noted to have improved, although right-sided pacing and circling persisted, and proprioceptive deficits in the left limbs remained. The first vaccine and imiquimod treatment were administered on January 14, 2019. Vaccinations continued at two-week intervals, with progressive neurological improvement. By the fifth vaccine, all neurological symptoms had resolved.

The patient experienced a brief seizure on March 1, 2019, which responded to an increase in prednisone and phenobarbital. Following stabilization, the patient resumed maintenance doses following initial stabilization. No further seizures occurred during vaccine treatment. On March 25, 2019, the patient presented for the sixth and final vaccine, but treatment was delayed due to a cough and diarrhea, which resolved with symptomatic therapy. The final vaccine was administered on April 1, 2019, at which time the neurological examination remained normal.

Attempts to taper prednisone, gabapentin, and phenobarbital followed. Seizure activity returned after dosage reduction, though episodes were brief and followed by rapid recovery. On May 6, 2019, phenobarbital was increased again, while tapering of gabapentin and prednisone continued. The final prednisone dose was administered at the end of May 2019.

A neurological exam on July 5, 2019, revealed recurrence of postural deficits in the left pelvic limb. After discontinuation of the vaccine series, seizures recurred approximately every 6–8 weeks. Based on these findings and the recommendation of Dr. Pluhar from the University of Minnesota, booster doses of the autologous lysate vaccine and imiquimod were administered. The owner noted a persistent cough and intermittent ataxia during this period. The second booster was administered on November 25, 2019. Thoracic radiographs were performed and were within normal limits for the patient’s age and breed. The third booster was given on January 20, 2020, with a normal neurological examination and no seizures reported since November 25, 2019.

Phenobarbital tapering was initiated on January 9, 2020, due to owner-observed side effect of lethargy. The patient’s disposition returned to the baseline prior to the onset of symptoms associated with the illness. However, two seizures occurred in March 2020, prompting the addition of levetiracetam on March 30, 2020. Neurological examination remained normal, although intermittent coughing persisted.

On July 8, 2020, the patient had one seizure between visits. Thoracic radiographs were repeated due to chronic coughing and revealed soft tissue masses throughout the lungs. The owner declined further diagnostics. Neurological findings were unchanged.

**Figure 2:**
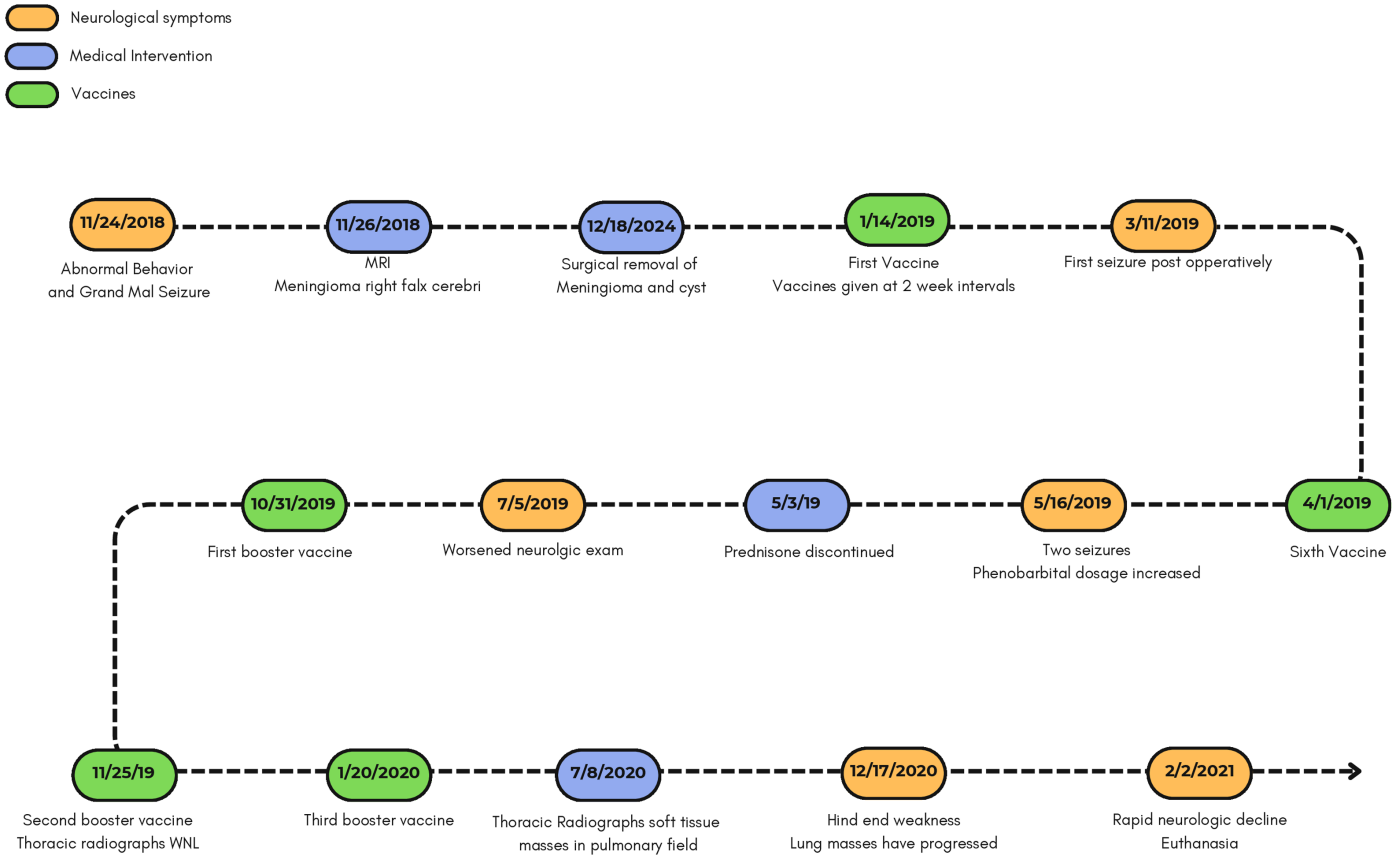
Timeline of neurological events and treatment from onset to euthanasia.

At a follow-up on September 25, 2020, neurological examination remained normal, although the cough persisted and labored breathing was noted. On December 17, 2020, the owner reported hindlimb weakness. Seizure activity remained minimal and appeared linked to missed anticonvulsant doses. Thoracic radiographs at that time showed progression of pulmonary masses. The patient relocated to Arizona in January 2021 and was euthanized on February 2, 2021, due to rapid neurological decline reported by the owner over the previous two weeks. A timeline of the patient’s history is shown in Figure 2.

**Figure 3:**
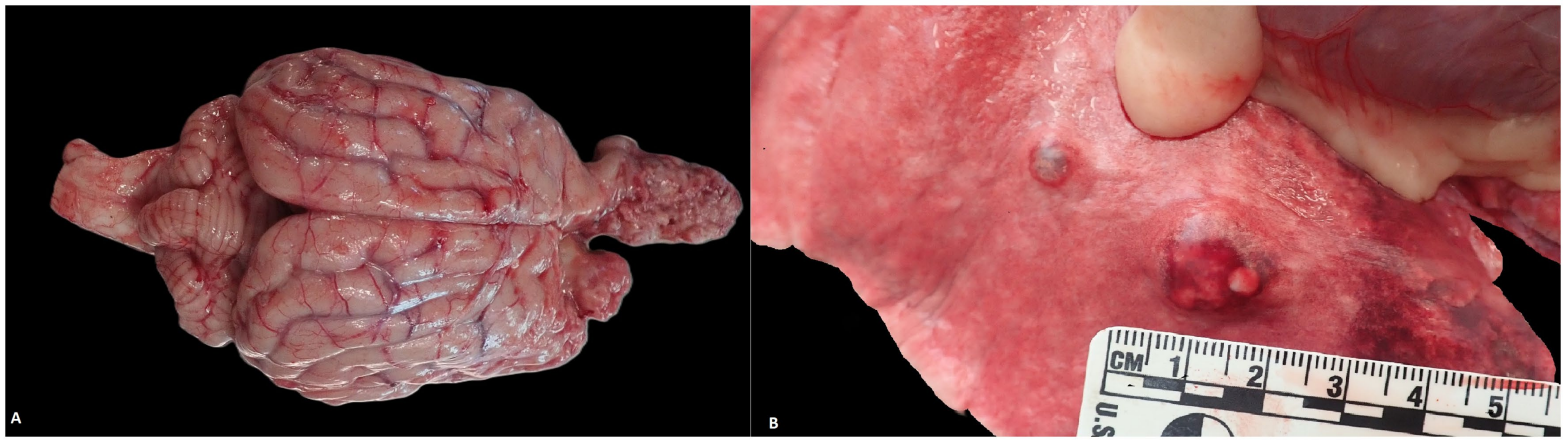
Macroscopic image of metastatic meningioma. **(A)** Primary meningioma extending from the frontal lobes of the brain. **(B)** Lungs containing multifocal raised metastatic meningiomas.

Necropsy was performed at Midwestern University Diagnostic Pathology Laboratory. The patient was in poor body condition. A focal area of missing frontal bone rostral to the sagittal crest was noted. Upon opening the skull, a flexible gray mesh was found beneath dense connective tissue and overlying a yellow-tan fibrous plate at the level of the frontal sinuses. Beneath this plate was a friable, white-tan mass extending from the olfactory bulbs into both nasal canals and turbinates (Figure 3A).

Six smooth, white-tan nodules were found in the left lung lobes; the right lung lobes were unaffected (Figure 3B). The abdominal cavity contained approximately 50 mL of serosanguinous fluid. Numerous firm white-tan nodules were present throughout the omentum. The liver was infiltrated with hundreds of multifocal, slightly raised white-tan masses. A large, multilobulated white-tan mass was found in the right limb of the pancreas (Supplementary Figure 2A), while the left limb appeared nodular. Similar nodules were found in the spleen, adrenal glands, kidneys, stomach, and omentum.

Impression smears of a lung mass showed high cellularity with mild to moderate blood contamination on a pale eosinophilic background. Spindle-shaped cells, found singly, in streaming aggregates, and occasional whorl-like formations, measured 7–9 μm in width and 50–80 μm in length (Supplementary Figure 1). Cells had moderate N: C ratios, mild to moderate anisocytosis and anisokaryosis, moderate pale basophilic cytoplasm (sometimes containing coarse magenta granules), and centralized oval nuclei with smooth to finely stippled chromatin, and 1–2 distinct nucleoli. Occasional binucleated and rare multinucleated cells were present, with rare mitotic figures. Focal lymphoplasmacytic and macrophagic infiltrates were noted, as well as rare columnar epithelial cell clusters. Findings supported a malignant neoplasm, with differential diagnoses of metastatic meningioma or sarcoma.

**Figure 4:**
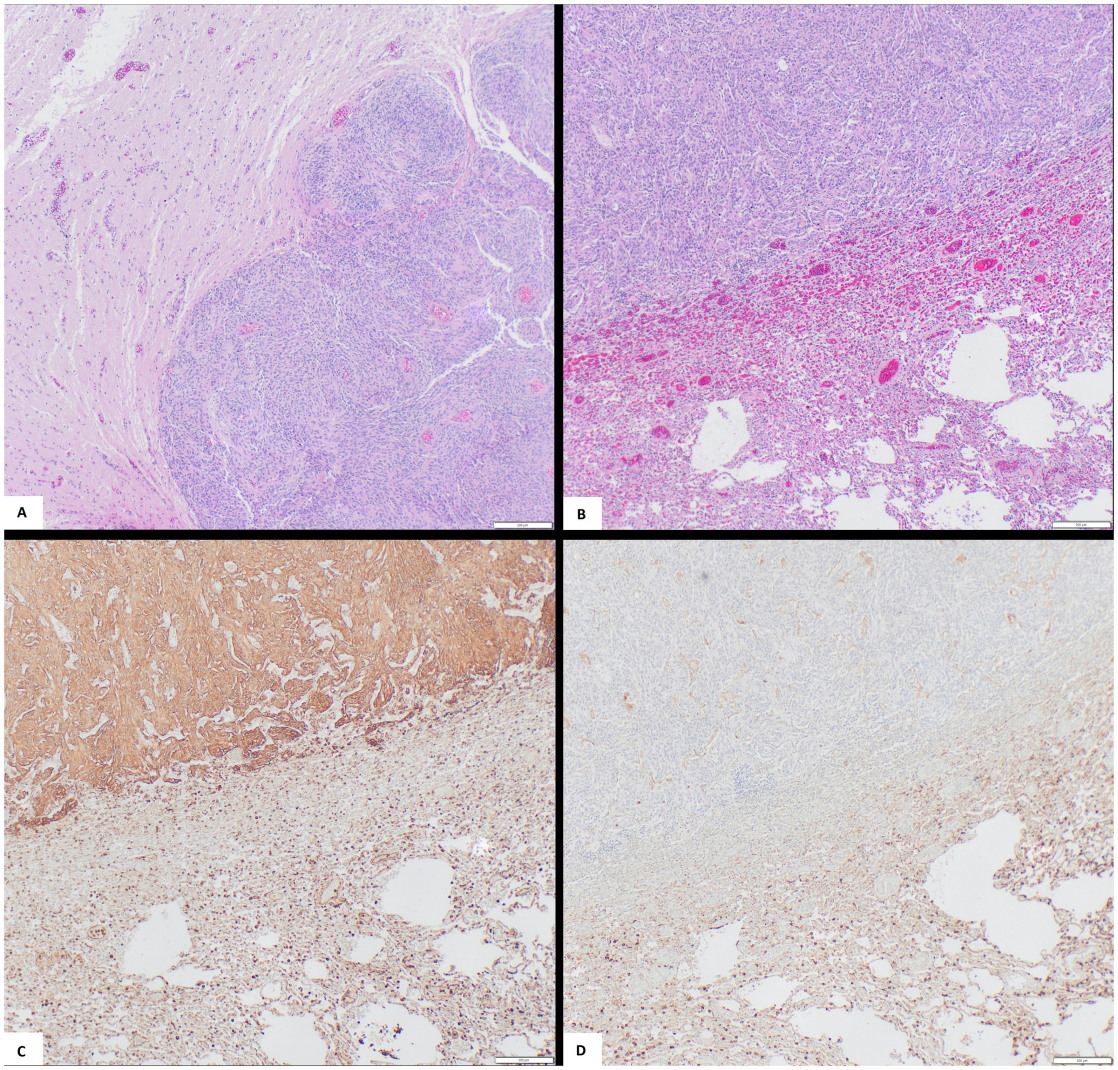
Microscopic panel of metastatic meningioma. **(A)** H&E of primary meningioma in the brain. **(B)** H&E of metastatic meningioma in the lungs. **(C)** Vimentin Immunohistochemistry with positive staining in lung metastasis. **(D)** Cytokeratin Immunohistochemistry with negative staining in lung metastasis.

Histopathology of the recurrent intracranial and nasal mass showed streams, whorls, and pseudorosettes of spindlyloid to polygonal cells with low mitotic activity, moderate to marked anisocytosis, and mild anisokaryosis, invading the olfactory bulbs (Figure 4A). Pulmonary masses exhibited similar morphology (Figure 4B). Compared to the original tumor, the recurrent intracranial mass had a lower mitotic index, more spindly cells, and clear evidence of tissue invasion.

Histopathology of the pancreatic mass revealed polygonal neoplastic cells in sheets, acini, and islands, with marked anisocytosis, moderate anisokaryosis, a high mitotic rate, and large areas of coagulative necrosis. Similar lesions were observed in the heart, liver, kidneys, spleen, adrenal glands, stomach, and small intestines (Supplementary Figure 2B).

Immunohistochemistry (IHC) was used to differentiate between metastatic meningioma and pancreatic adenocarcinoma in the lungs. Vimentin showed strong positive staining in 60–80% of neoplastic cells in the lungs (Figure 4C) and intracranial mass. Cytokeratin showed mild to moderate reactivity in 60–70% of pancreatic tumor cells (Supplementary Figure 2B), 10–15% of liver cells, and none of the pulmonary neoplastic cells (Figure 4D). S100 was negative with nonspecific background staining in all tissues.

Based on histopathological and IHC findings, the patient was diagnosed with a recurrent and metastatic intracranial meningioma with pulmonary involvement, as well as a separate pancreatic carcinoma with widespread metastases.

## Discussion

Meningiomas are the most common intracranial tumors in both dogs and humans (1); however, metastasis is extremely rare, occurring in fewer than 1% of human cases (2), and with only four reported cases of metastatic meningioma in dogs (3, 4). The majority of human meningiomas are considered benign, though recurrence is possible after resection (9). In human medicine, recurrence after resection and classification as grade II or higher appear to be the greatest risk factors for metastatic disease (2). In canines, the median survival time has been previously reported as 16.5 months with treatment (5), which is shorter than the average disease course in humans diagnosed with metastatic disease (2). This shorter survival time in dogs may explain the rarity of metastatic meningioma diagnoses in canine patients.

Treatments that have demonstrated significant improvements in survival time typically involve surgical resection and/or radiation therapy, with reported median survival often ranging from 7 to 22 months depending on the study (5–7). There is one study reporting a notably longer median survival of 2,104 days for a subset of meningioma patients with endoscopic surgery (8); however, it is difficult to compare between studies due to variability and sometimes lack of clarity in inclusion/exclusion criteria. In this case, the patient received immunotherapy in combination with surgical treatment and achieved a survival duration of 28 months—exceeding the median survival times reported for most conventional treatment modalities.

Canine meningiomas closely mimic human disease progression and development at both histological and genetic levels (11, 12). One key difference is the higher recurrence rates and longer survival times observed in human patients (2). The extended disease course and recurrence of the tumor in this case mirror the conditions that increase metastatic risk in human patients (2). This may suggest that metastatic progression in the case may be related to length of extended survival time rather than tumor behavior alone.

The patient’s advanced age was likely responsible for the concurrent pancreatic adenocarcinoma, as age is the most significant risk factor associated with the development of this cancer (13, 14). The pancreatic adenocarcinoma presented in a typical manner (14), further supporting the conclusion that it developed independently from the meningioma. Two dogs in the original experimental cohort of 11 meningioma patients receiving autologous vaccines also developed concurrent cancers. One developed lymphoblastic lymphoma and was euthanized 25 weeks after final vaccine and the other died due to an unknown abdominal tumor 2.4 weeks after final vaccine (10). A more recent study using similarly manufactured immunotherapy vaccines in a cohort of dogs with gliomas found no cases of secondary cancers at time of death (15). Given the low number of reported outcomes in patients treated with this canine immunotherapy, it is difficult to say whether there is a link between treatment and additional cancer development. Human trials of autologous immunotherapy vaccines have reported a possible increase in secondary hematological malignancies, such as lymphoma, but not higher cases of other secondary cancers (16, 17). The type of secondary cancer in this patient is more suggestive of contribution by external risk factors, such as advanced age (13, 14); however, differences between human and canine responses to immunotherapy are not fully understood.

The canine case described in Ortiz-Nisa et al. (3) involved a more aggressive malignant meningioma. Its histopathological and immunohistochemical (IHC) features differed from this case and others previously published, showing characteristics such as a high mitotic rate and fusiform-shaped cells (3, 4). Additionally, in that case, the lung metastasis was larger than the suspected primary intracranial meningioma (3), which contrasts with this and other described cases (4). The three metastatic meningioma cases described by Schulman et al. (4) shared more histologic similarities with this case, most notably a low mitotic index in both primary and metastatic tumors (4). One hypothesis is that a low mitotic index may allow the tumors to grow slowly, avoiding rapid neurological deterioration and giving the tumor time to metastasize (18).

The 2022 case stained positive for vimentin but differed from this case in showing strong S100 positivity as well (3). Still, the IHC stain findings in this case align with the typical meningioma staining patterns: strong vimentin positivity in both the primary and metastatic tumors (19, 20), confirming meningioma metastasis to the lungs. The use of vimentin, which strongly stained this meningiomas, alongside cytokeratin, which did not stain this meningioma, allowed clear differentiation between the two neoplastic populations in the body.

The field of veterinary immunotherapy lags behind human medicine (21, 22). Major reasons include the high cost of immunotherapies, which are often unaffordable for pet owners, as well as, a lack of external funding for veterinary research (21). Our understanding of canine immunological reagents is far behind human medicine, and the absence of centralized databases and large-scale collaboration further hampers progress (23). However, there have been promising advancements. These include autologous vaccines like the one used in this case (10), as well as in a recent clinical trial in treating osteosarcoma (24). Immune checkpoint inhibitors (ICIs), though still underdeveloped in veterinary medicine (25), have been used in clinical trials. As of late 2023, the first USDA-conditionally licensed ICI for treating canine neoplasia became commercially available, with additional products in development (22). Given these trends, the future of immunotherapy in veterinary oncology—particularly for canine patients—seems to be on the rise and may assume a more prominent role in clinical practice in the future.

This case highlights the impact that emerging immunotherapies can have on patient outcomes, especially given the fact this patient seizure activity lessened when boostered with additional immunotherapy vaccines. This dog lived 6 months longer than the previously reported median survival time for dogs treated with conventional meningioma therapies (5). While this case may represent an outlier, it supports the potential of this treatment modality and further investigation in a larger cohort. Additionally, this patient had a severe concurrent neoplasia that likely contributed to his decline and ultimate euthanasia. Although the metastatic progression is concerning, its cause remains unclear and may be attributed to the prolonged disease course rather than the autologous vaccine or adjuvant therapy. Nonetheless, the survival time observed in this case, along with ongoing advancements in canine oncology, offers continued hope for extending the lives of dogs diagnosed with cancer. Immunotherapy shows promise as an evolving and impactful approach in both veterinary and human medicine.

## Data Availability

The original contributions presented in the study are included in the article/Supplementary material, further inquiries can be directed to the corresponding author/s.

## Supplementary material

SUPPLEMENTARY FIGURE 1 Impression cytology of the lung lesion. There are numerous spindle shaped cells that are found individually and in aggregates. The cells have variably distinct cellular borders and often elongated cytoplasmic projections. Occasionally whirl- like formations are seen (arrowhead). Wright-Giemsa.

SUPPLEMENTARY FIGURE 2 Panel of primary pancreatic carcinoma. **(A)** Macroscopic image of primary pancreatic carcinoma and hepatic metastasis. **(B)** Microscopic images of H&E of primary pancreatic carcinoma **(C)** Cytokeratin Immunohistochemistry with positive staining of pancreatic carcinoma.
