# Supplementary figures and images for: The development of metastatic meningioma in a canine patient post-immunotherapy case report
Source: Front Vet Sci. 2026 Jan 5;12:1646793. doi: 10.3389/fvets.2025.1646793 (PMC12812729; doi:10.3389/fvets.2025.1646793)

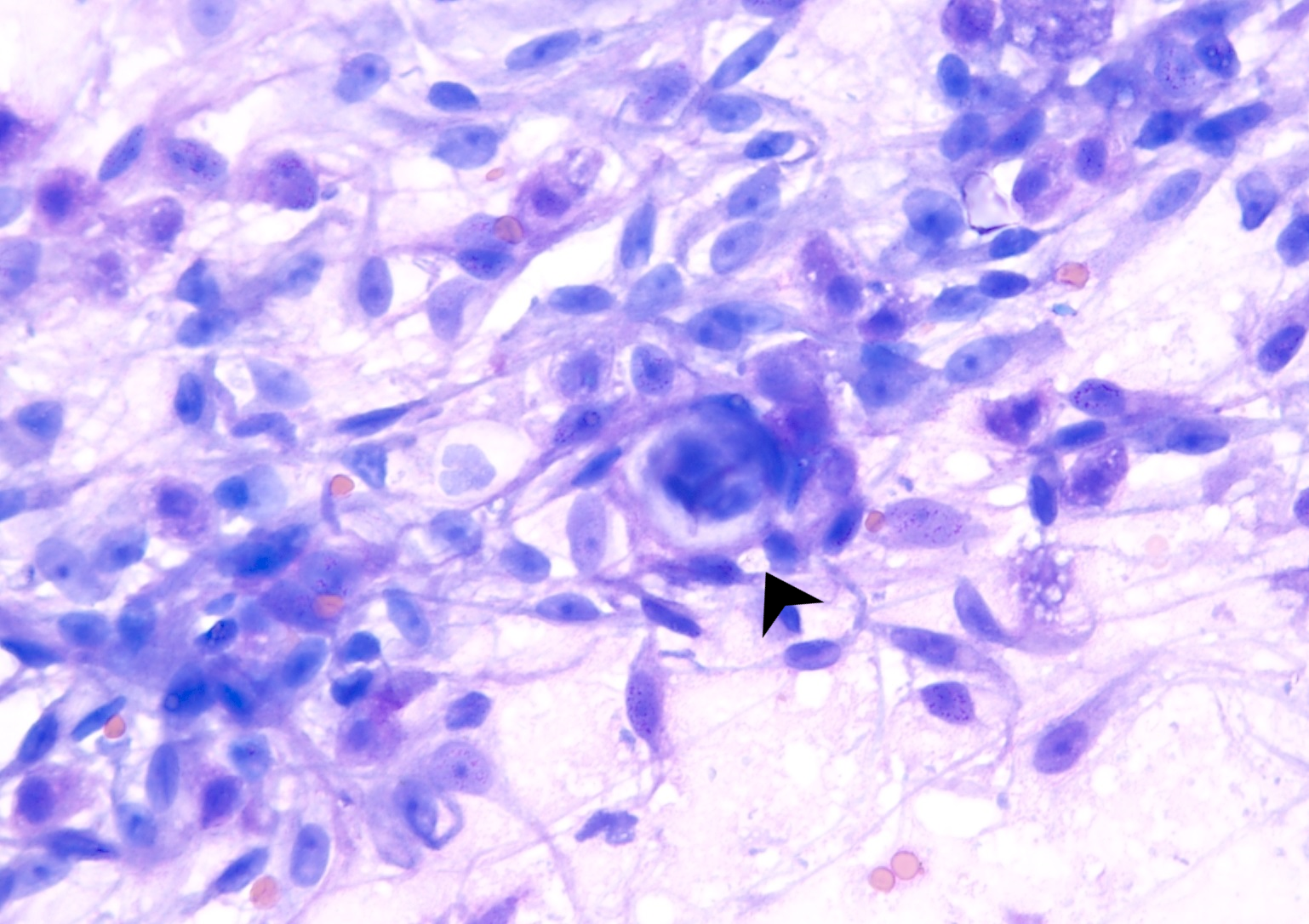

Supplement: SUPPLEMENTARY FIGURE 1 — Impression cytology of the lung lesion. There are numerous spindle shaped cells that are found individually and in aggregates. The cells have variably distinct cellular borders and often elongated cytoplasmic projections. Occasionally whirl- like formations are seen (arrowhead). Wright-Giemsa. [file Image_1.TIFF]

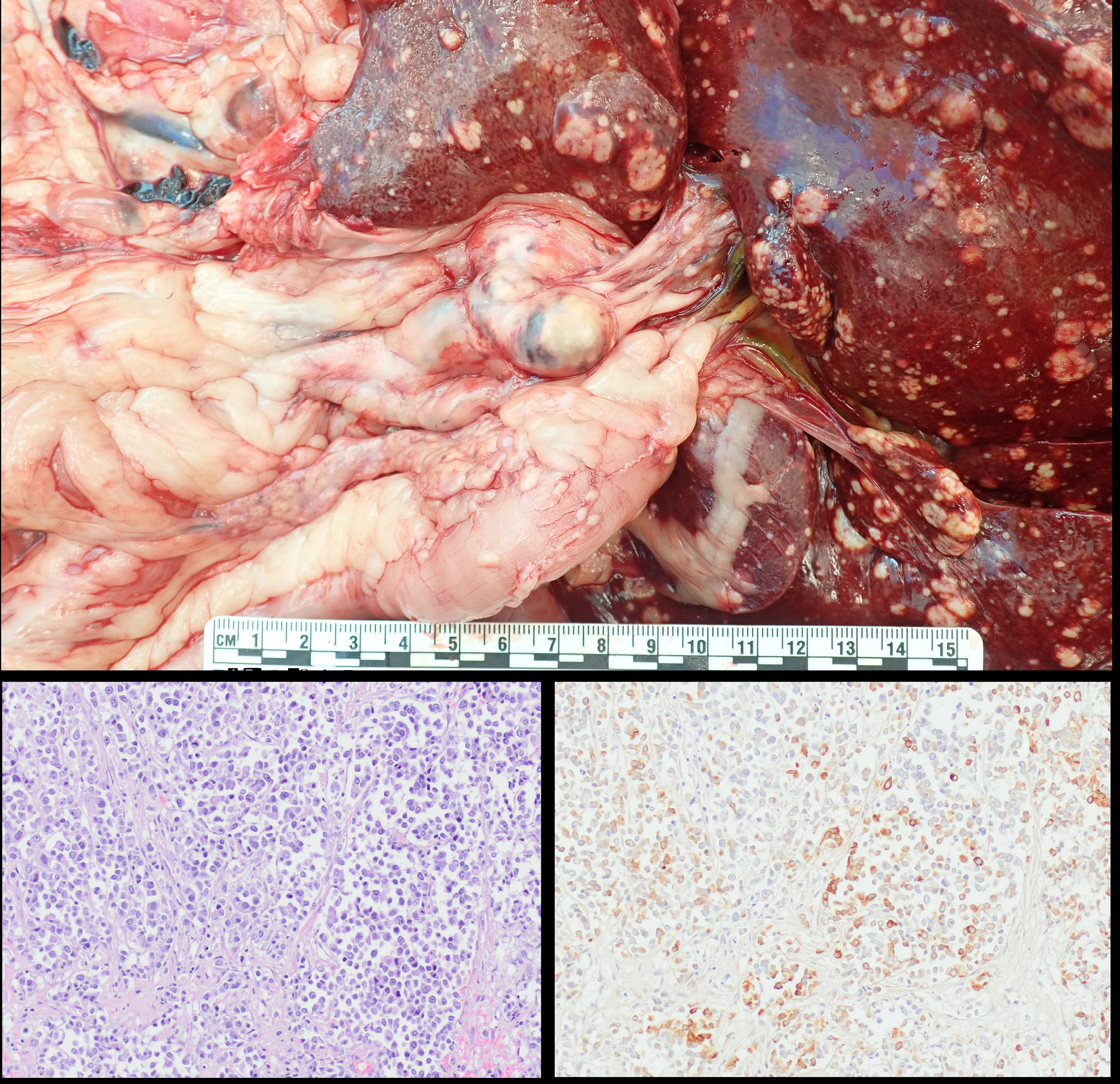

Supplement: SUPPLEMENTARY FIGURE 2 — Panel of primary pancreatic carcinoma. (A) Macroscopic image of primary pancreatic carcinoma and hepatic metastasis. (B) Microscopic images of H&E of primary pancreatic carcinoma (C) Cytokeratin Immunohistochemistry with positive staining of pancreatic carcinoma. [file Image_2.TIF]
